# Prognostic significance for colorectal carcinoid tumors based on the 8th edition TNM staging system

**DOI:** 10.1002/cam4.3431

**Published:** 2020-09-08

**Authors:** Piqing Gong, Chunhua Chen, Zhan Wang, Xukun Zhang, Wenxin Hu, Zhiqian Hu, Xinxing Li

**Affiliations:** ^1^ Department of General Surgery Changzheng Hospital the Second Military Medical University Shanghai China; ^2^ Department of the VIP section Changzheng Hospital the Second Military Medical University Shanghai China; ^3^ Department of Oncology Changzheng Hospital the Second Military Medical University Shanghai China; ^4^ School of Data Science & Engineering East China Normal University Shanghai China

**Keywords:** colorectal carcinoid, neuroendocrine carcinoma, prognosis, SEER, TNM stage

## Abstract

The aim of our study was to explore the value of the 8th edition TNM staging system on evaluating the prognosis of colorectal carcinoid. Colorectal carcinoid patients between 1988 and 2015 were selected in the Surveillance, Epidemiology, and End Results Program (SEER) database for analysis. About 4286 patients with colorectal carcinoid tumors were identified, of which were carcinoid tumor NOS (n = 1726), neuroendocrine carcinoma (NEC) (n = 1346) and other carcinoid tumor (OCT) (n = 591). Worsening 10‐year CSS rates with increasing N status, M status, and SEER historic stage were demonstrated across all three above groups (all *P* < .05). In carcinoid tumor NOS, significant differences in CSS were found with increasing combined 8th AJCC stages (*P* < .001), except for that between stage II and stage III (10‐year CSS rate: 82.6% vs 84.3%, *P* = .68). While combined 8th TNM stage in NEC and OTC exhibited greater separations in CSS despite on‐going overlaps between groups. For carcinoid tumor NOS, stage II (HR = 3.37; 95% CI: 0.97‐11.76), and stage III (HR = 2.09; 95% CI: 0.51‐8.66) conferred no significant difference in CSS compared with stage I, while stage IV had an increasing HR of 5.09 (95% CI: 1.08‐24.08). Although combined 8th AJCC stage had a good ability to distinguish 10‐year CSS of patients with NEC or OCT, detailed 8th AJCC stage did not seem to be applicable. Detailed 8th AJCC categories of advanced stages in all the three groups conferred increased HRs with overlapping CIs. However, in the early and middle status, HRs did not increase with the increase of stages, or there was no difference in HRs between adjacent stages. Combined 8th TNM stage was not practical for judging the survival outcomes of colorectal carcinoid tumor NOS, especially in patients with stages II and III, but it provided useful prognostic information for NEC and OCT. However, for all carcinoid tumors, the prognostic values of detailed 8th AJCC stage were not enough accurate in the clinic. More optimized staging methods should be developed and validated in the future.

## Graphical abstract

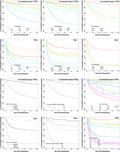

## 1 INTRODUCTION

Carcinoid tumors, first described by Langhans, were slow‐growing neoplasms that originated from neuroendocrine cells, marked by endocrine properties, and a special phenotype that had been re‐recognized and studied in most organs throughout the body, including gastrointestinal tracts.^1^, ^2^ However, these carcinoids show different types and clinical outcomes in the process from benign to malignant.^3^ As the screening popularity of colonoscopy, colorectal carcinoids were more commonly found in the United States, with rectal carcinoids accounting for about 20%.^4^, ^5^ Because of the lack of uniform classification standards and relatively special nature, no recognized and unified staging system existed for these tumors.^6^ The World Health Organization (WHO) Classification of the Digestive System 2010 used “neuroendocrine neoplasm (NEN)” to bridge this classification gap.^2^ But Yozu et al^7^ reclassified appendiceal Goblet cell carcinoids not based on the NEN staging system, and furthermore found its grading and staging was similar to colonic adenocarcinomas. The 8th edition TNM staging system redefined staging criteria for colorectal and rectal neuroendocrine tumor (NET), with grade G1/G2 or well‐differentiated grade G3 using T, N, and M descriptors after their adenocarcinoma counterparts. Researchers had evaluated the prognostic validity of these new NET staging systems with mixed results.^8^ Further, the 8th edition of the American Joint Cancer Committee (AJCC) stage stipulated that the colorectal cancer stage was applicable to adenocarcinoma, neuroendocrine carcinoma (NEC), and squamous cell carcinoma.

To our knowledge, most of the studies did not distinguish between colorectal NEC and non‐NEC when evaluating the prognostic outcome of carcinoid tumors, and also validation on practicality and accuracy for carcinoid tumors according to the 8th TNM stage had not yet been explored. In our cohort, population‐based data from a national cancer registry were selected to evaluate the value of the 8th AJCC staging system on the prognosis of colorectal carcinoid.

## 2 METHODS

### 2.1 Data source and study parameter

A retrospective cohort study from 1998 to 2015 was conducted to select patients, diagnosed with colorectal carcinoid tumor, whose age more than 18 years in the Surveillance, Epidemiology, and End Results (SEER) database. This version of the SEER database we used had been released April 2018 (November 2016 submission). Cases were stratified by histopathological type as follows: carcinoid tumor NOS (8240/3), NEC (8241/3), and OCT (8243/3, 8244/3, 8245/3, 8246/3, and 8249/3). Tumor sites were limited to colon (C18.0‐18.9) and rectum (C19.9 and C20.9). The included subjects contained a single primary malignancy and clear follow‐up information. Age, sex, race, tumor location, grade, histologic type, marital status, T status, N status, M status, and surgery information were assessed. TNM classification was restaged according to the 8th edition AJCC Cancer Staging Manual. Because in SEER data N1c (tumor implantation in subserous, mesenteric or non‐peritoneal‐covered, without regional lymph node metastasis) was missing, no analysis of the N1c subgroup was performed. Because no obvious distinctions of M1b (metastasis distributed in more than one organ) and M1c (peritoneal metastasis with or without other organ metastasis) were made, stages IVB and IVC were merged for analysis as stage IV_B+C_. Although invasive depth (Tx) or positive lymph node metastasis (Nx) was unknown, some tumors had been confirmed to show distant metastasis (M1). Thus, these data were still included in the study, staging as stage IV (IVA, IV_B+C_ or IVx), according to the actual status of tumor metastasis.

### 2.2 Statistical analysis

In order to facilitate the statistical calculation, we combined goblet cell carcinoid, mixed adenoneuroendocrine carcinoma, adenocarcinoid tumor, enterochromaffin cell carcinoid, and atypical carcinoid tumor into other carcinoid tumors (OCT). The main outcome was death from colorectal carcinoid tumor. The cumulative incidence function (CIF) method, described by Pepe and Mori,^9^ was used to compared cancer‐specific survival (CSS) by stage. Ten‐year CSS was estimated via calculating the complement of the cumulative incidence of death due to colorectal carcinoid tumor. Competing events were seen as deaths of other causes other than colorectal carcinoid tumors. Then we plotted the curves comparing groups of interest by 1‐CIF. The Fine‐Gray method was used for multivariable CIF competing risks analyses after adjusting variables. All statistical analyses were performed by the R version 3.1.5 (http://www.R‐project.org/). Two‐sided *P* value less than .05 was considered significant.

## 3 RESULTS

### 3.1 Patient characteristics

As shown in Table S1, 4286 colorectal carcinoid patients were identified. In this cohort, women and whites accounted for 52.7% and 79.4%, respectively, and the average age was 58 years old (SD, 14.4 years). Most tumors located in colon (84.0%), and 88% of all cases received surgery. There were more cases with stage IV (35.9%), T3 stage (43.2%) or N+ (47.8%) in all cases. The pathological types of the patients we selected to join the study were as follows: carcinoid tumor NOS (40.2%), NEC (31.4%), goblet cell carcinoid (13.8%), mixed adenoneuroendocrine carcinoma (8.0%), adenocarcinoid tumor NOS (5.5%), enterochromaffin cell carcinoid (0.19%), and atypical carcinoid tumor NOS (0.9%). The latter five pathological types were combined into OCT (28.4%). Sun‐stages from IA to IVx accounted for 8.7%, 4.2%, 17.3%, 2.1%, 1.1%, 5.4%, 17.3%, 7.9%, 2.1%, 22.9%, and 11.0% of patients, respectively.

### 3.2 Analysis of 10‐year CSS rate in carcinoid tumor NOS

**FIGURE 1:**
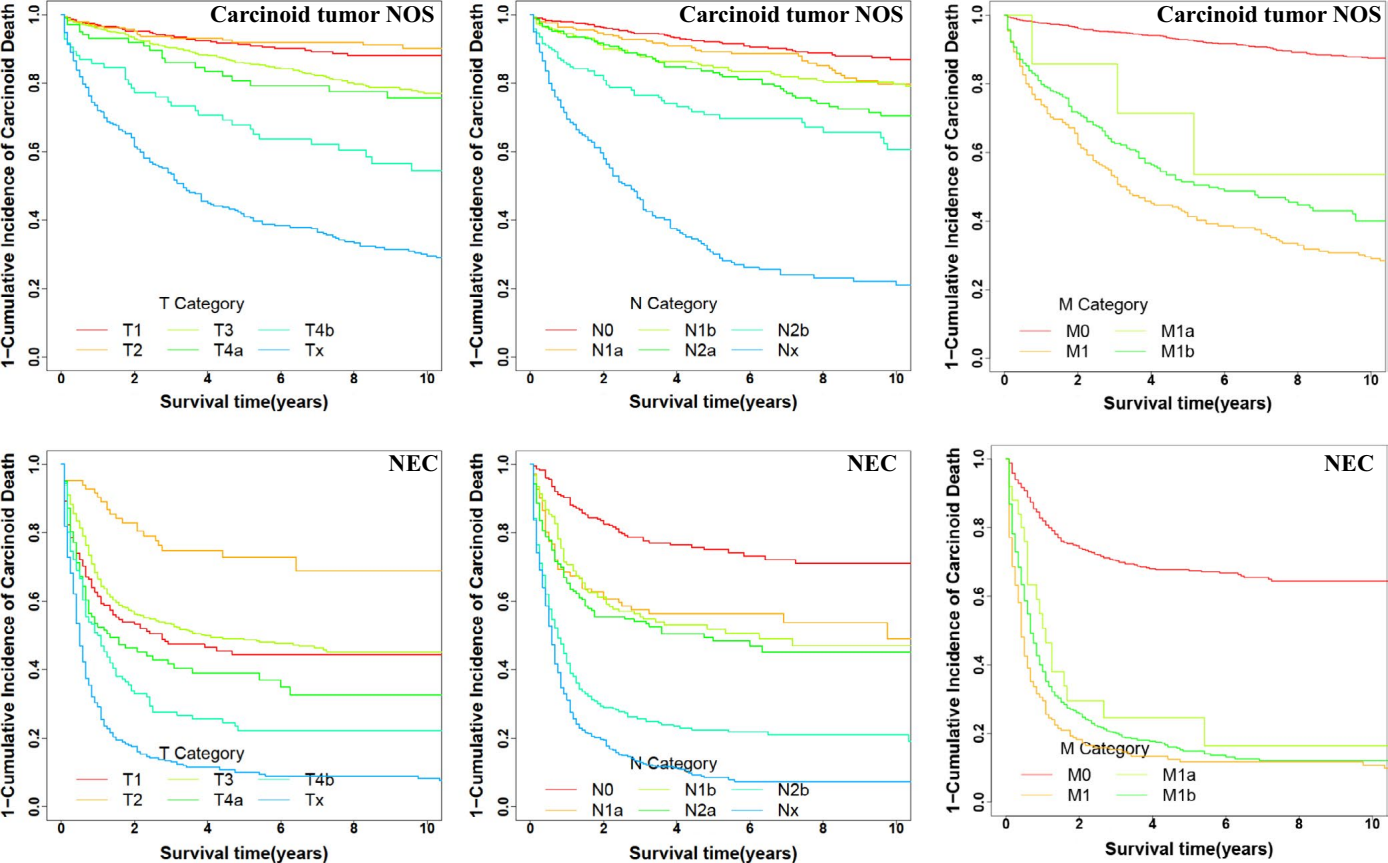
Cumulative incidence function curves for 10‐y CSS. The 1 minus cumulative incidence of carcinoid death for carcinoid tumor NOS and NEC by T status, N status, and M status

Within carcinoid tumor NOS (n = 1726), T stage was an important indicator for CSS (*P* < .001), except for differences in CSS between stages T1 and T2 (*P* = .594) and between stages T3 and T4a (*P* = .670) (Figure 1; Data S1). Stages T1, T2, and T3 exhibited 10‐year CSS rates of 88%, 90.2%, and 76.9%, respectively. T4a/T4b/T_x_ showed CSS rates of 75.6%/54.5%/29.5%. Marked differences in CSS were demonstrated with increasing N stage (*P* < .001), except for that in CSS between N1a and N1b stages (*P* = .479). N0 stage had a CSS rate of 86.9% compared with that for N1a/N1b, N2a/N2b, and Nx (rates at 10 years of 79.8%/79.7, 70.4%/60.5 and 21.0%, respectively). Also, M stage was an obvious indicator for CSS (*P* < .001). CSS declined steeply to 29.0% for M1, compared with M0 (87.4%). However, M1a had a similar 10‐year rate of CSS with M1b (53.6% vs 40.1%, *P* = .472). Marked differences in CSS were showed with increasing SEER historic stage (*P* < .001), and combined 8th AJCC stages (*P* < .001) except for differences in CSS between stage II and stage III (10‐year CSS rate: 82.6% vs 84.3%, *P* = .68).

**FIGURE 2:**
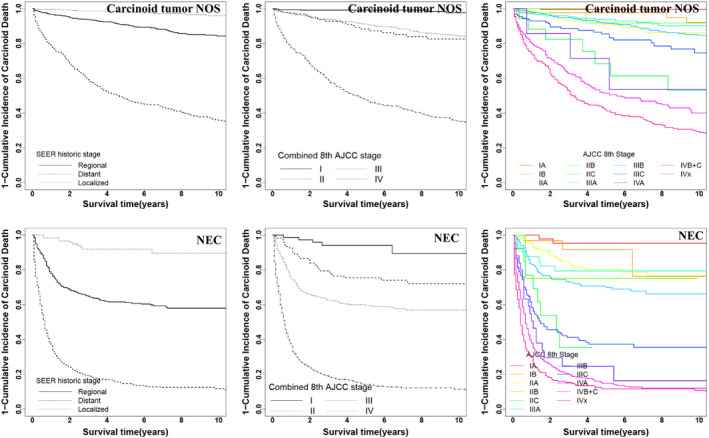
Cumulative incidence function curves for 10‐y CSS. The 1 minus cumulative incidence of carcinoid death for carcinoid tumor NOS and NEC by SEER historic stage, combined 8th AJCC stage, and detailed 8th AJCC stage

Overall, detailed 8th AJCC stage demonstrated marked differences in CSS (*P* < .001), except for those between IIA and IIC (10‐year CSS rate: 86.2% vs 91.7%, *P* = .132), between IIA and IIIA (10‐year CSS rate: 86.2% vs 90.1%, *P* = .222), between IIB and IIIA (10‐year CSS rate: 91.7% vs 90.1%, *P* = .653), between IIB and IIIB (10‐year CSS rate: 91.7% vs 84.6%, *P* = .063), and between IIC and IIIC (10‐year CSS rate: 53.1% vs 75.4%, *P* = .386). The rates of 10‐year survival decreased to 53.6% and 40.1% for stages IVA and IV_B+C_, respectively, with no significant difference between them (*P* = .175). Besides, the rates of 10‐year survival were 99.6% and 92.1% for stages IA1 and IB, respectively (*P* = .005) (Table 1; Figure 2, Data S1).

**TABLE 1:** Ten‐year CSS

TNM category	Carcinoid tumor NOS			NEC			OCT		
	N	10‐y survival, %	95% CI	n	10‐y survival, %	95% CI	n	10‐y survival, %	95% CI
*SEER historic stage*									
Localized	445	95.9	92.9‐97.9	121	89.6	81.6‐95.1	486	91.4	87.7‐94.3
Regional	785	84.2	81.0‐87.2	437	58.0	52.9‐63.2	439	62.9	57.3‐68.6
Distant	496	35.7	31.2‐40.7	788	11.8	9.2‐15.1	289	7.2	4.3‐12.0
*Combined 8th AJCC stage*									
I	336	97.5	94.2‐99.2	76	89.5	76.3‐97.1	141	96.8	91.5‐99.2
II	200	82.6	75.1‐89.0	120	72.1	63.0‐80.6	560	87.9	84.1‐91.2
III	710	84.3	80.9‐89.4	369	56.9	51.4‐62.6	236	39.4	32.1‐47.8
IV	480	35.2	30.6‐40.3	781	11.6	9.0‐14.8	277	6.4	3.6‐11.2
*T stage*									
T1	388	88.0	83.9‐91.5	158	44.4	36.6‐53.0	90	85.4	76.1‐92.5
T2	235	90.2	85.4‐94.0	83	68.9	56.4‐80.6	82	88.4	78.6‐95.1
T3	665	76.9	72.7‐80.9	542	45.2	40.6‐50.1	639	74.5	70.3‐78.6
T4a	101	75.6	65.5‐84.6	101	32.7	23.3‐44.6	173	48.9	39.9‐58.9
T4b	84	54.5	43.1‐66.6	126	22.3	15.6‐31.3	117	23.8	16.1‐34.2
Tx	253	29.5	24.0‐35.9	336	8.3	5.5‐12.3	113	6.1	2.6‐14.2
*N stage*									
N0	584	86.9	83.1‐90.2	228	71.0	64.1‐77.7	736	87.1	83.9‐90.0
N1a	250	79.8	73.4‐85.5	111	49.0	36.8‐62.9	102	45.0	33.8‐58.0
N1b	296	79.7	74.4‐84.5	151	47.1	38.3‐56.8	99	27.1	17.2‐41.1
N2a	230	70.4	63.2‐77.4	176	45.1	37.0‐54.1	87	20.5	12.3‐33.0
N2b	166	60.5	51.1‐70.1	285	21.0	16.3‐26.7	98	8.7	4.0‐18.6
Nx	200	21.0	15.2‐28.5	395	7.4	4.9‐11.1	92	0^a^	—
*M stage*									
M0	1246	87.4	85.0‐89.6	565	64.4	60.0‐68.9	937	77.3	73.9‐80.5
M1a	7	53.6	19.8‐93.1	25	16.4	4.5‐50.2	56	0^a^	—
M1b	278	40.1	33.3‐47.6	555	12.1	9.1‐15.8	147	0^a^	—
M1	195	29.0	23.0‐36.2	201	10.8	6.9‐16.7	74	5.8	2.0‐16.3

Abbreviations: CI, confidence interval; CSS, cancer‐specific survival. OCT: goblet cell carcinoid, mixed adenoneuroendocrine carcinoma, adenocarcinoid tumor, enterochromaffin cell carcinoid, and atypical carcinoid tumor.

^a^ No events occurred in this group.

### 3.3 Analysis of 10‐year CSS rate in NEC and OCT

For both NEC (n = 1346) and OCT (n = 1214), significant differences in CSS were found with increasing SEER historic stage (localized, regional and distant, all *P* < .001), combined 8th AJCC stage (*P* < .001), and increasing M status (M0 and M1, *P* < .001). Although, T stage was also an important indicator for CSS (*P* < .001) both in NEC and in OCT, again showing overlaps in CSS curves between T stages. The 10‐year CSS rate of T1 was less than that of T2 (44.4% vs 68.9%, *P* < .001); however, similar to than that of T3 (44.4% vs 45.2%, *P* = .306) and T4a (44.4% vs 32.7%, *P* = .201) in NEC. Besides, there was no difference in the 10‐year CSS rate between T4a and T4b (32.7% vs 22.3%, *P* = .072). In OCT, the rate of 10‐year survival of T1 was similar that of T2 (85.4% vs 88.4%, *P* = .19) and T3 (85.4% vs 74.5%, *P* = .145). With the progression of the N stage from N0 to N2b, the 10‐year CSS rates decreased gradually both in NEC and in OCT. But in NEC, the 10‐year CSS rates of N1a, N1b, and N2a overlapped (*P*_N1a vs N1b_ = .745, *P*_N1a vs N2a_ = .267 and *P*_N1b vs N2a_ = .399), while in OCT N1b and N2a overlapped (*P*_N1b vs N2a_ = .125) (Table 1, Figure 1; Figure S1, Data S1).

Although combined 8th AJCC stage had a good ability to distinguish the 10‐year CSS of patients with NEC and OCT, detailed 8th AJCC stage did not seem to be applicable. For example, the 10‐year CSS rate of the later stage was worse than that of the previous one, such as IB and IA (100% vs 94.0%, *P* = .019) in OCT. Even there was no difference in the 10‐year CSS rates of several stages, such as IB, IIA, and IIIA (76.3% vs 76.6% vs 79.4%; *P*_IB vs IIA_ = .311, *P*_IB vs IIIA_ = .286, and *P*_IIA vs IIIA_ = .851) in NEC, and IIB, IIC, and IIIA (75.2% vs 64.5% vs 58.1%; *P*_IIB vs IIC_ = .153, *P*_IIB vs IIIA_ = .184, and *P*_IIC vs IIIA_ = .079) in OCT (Table 1, Figure 2; Figure S1, Data S1).

### 3.4 HRS for cancer‐specific mortality in carcinoid tumor NOS

For carcinoid tumor NOS, T2 (HR = 1.09; 95% CI: 0.64‐1.86) showed no obvious difference in CSS compared with T1 stage, T1, T3, T4a, T4b, and Tx conferred HRs of 2.53 (95% CI: 1.68‐3.8), 2.80 (95% CI: 1.55‐5.05), 5.01 (95% CI: 3.02‐8.31), and 7.86 (95% CI: 5.11‐12.11), respectively, with overlaps between adjacent categories. All N categories had increasing HR values compared with N0 (N1b [HR = 1.52, 95% CI: 1.02‐2.27], N2a [HR = 2.16; 95% CI: 1.45‐3.20], N2b [HR = 2.65; 95% CI: 1.73‐4.05], and Nx [HR = 4.79; 95% CI: 3.01‐7.64]), except for N1a (HR = 1.25, 95% CI: 0.81‐1.92), with overlaps between adjacent stages. Compared with M0, M1a, and M1b conferred significant differences in CSS (HR = 3.36, 95% CI: 1.38‐8.61; HR = 5.19, 95% CI: 3.97‐6.79), showing overlaps between adjacent stages. Also compared with M0, M1 conferred a significant difference in CSS (HR = 7.74, 95% CI: 5.91‐10.14). The HR estimates of SEER regional and distant stages revealed a clearer pattern, showing HRs of 3.38 (95% CI: 1.64‐6.98) and 8.96 (95% CI: 3.21‐25.09), respectively, in comparison with localized. Stage IV had a significant pattern, conferring HRs of 5.09, compared with stage I, while stage II and stage III not (Table 2).

**TABLE 2:** Ten‐year CSS

TNM category	Carcinoid tumor NOS			NEC			OCT		
	N	10‐y survival, %	95% CI	n	10‐y survival, %	95% CI	n	10‐y survival, %	95% CI
*Detailed 8th AJCC stage*									
IA	252	99.6	97.8‐99.9	46	95.4	86.1‐99.2	74	94.0	84.8‐98.5
IB	84	92.1	81.5‐97.8	30	76.3	42.3‐97.7	67	100.0^a^	—
IIA	171	86.2	78.2‐92.4	103	76.6	67.0‐85.2	469	91.3	87.7‐94.2
IIB	12	91.7	67.7‐99.6	4	0^b^	—	72	75.2	60.8‐87.5
IIC	17	53.1	29.5‐80.8	13	0^b^	—	19	64.5	41.3‐86.6
IIIA	174	90.1	84.4‐94.4	40	79.4	65.5‐90.5	19	58.1	30.2‐87.8
IIIB	407	84.6	80.0‐88.6	201	66.2	58.6‐73.7	136	46.6	36.4‐58.1
IIIC	129	74.5	64.5‐83.6	128	35.5	27.3‐45.4	81	21.4	12.6‐35.2
IVA	7	53.6	19.8‐93.1	25	16.4	4.5‐50.2	56	0^b^	—
IVB + C	278	40.1	33.3‐47.6	555	12.1	9.1‐15.8	147	0^b^	—
IVx	195	29.0	23.0‐36.2	201	10.8	6.9‐16.7	73	5.8	2.0‐16.3

Abbreviations: CI, confidence interval; CSS, cancer‐specific survival; NEC, neuroendocrine carcinoma; OCT, other carcinoid tumor.

^a^ All members of this group had events.

^b^ No events occurred in this group.

CSS showed no obvious differences from the reference (stage IB) for stages IIA (HR = 2.08; 95% CI: 0.68‐6.38), IIB (HR = 1.22; 95% CI: 0.13‐11.43), IIIA (HR = 1.66; 95% CI: 0.55‐5.00) or IIIB (HR = 2.49; 95% CI: 0.89‐6.95), while stage IA revealed a smaller HR (HR = 0.087, 95% CI: 0.010‐0.788). In addition to those stages, HR estimates generally manifested an upward trend, with overlapping CIs (for stage IIIC, HR = 3.28 and 95% CI: 1.12‐9.65; for stage IVA, HR = 7.80 and 95% CI: 1.79‐34.00; for stage IV_B+C_, HR = 10.16, and 95% CI: 3.68‐28.00 and for stage IVx, HR = 14.97 and 95% CI: 5.44‐41.19) (Table 3).

**TABLE 3:** Adjusted CSS and cumulative incidence function regression models

TNM category	Carcinoid tumor NOS		NEC		OCT	
	HR (CIF)	95% CI	HR (CIF)	95% CI	HR (CIF)	95% CI
*Detailed 8th AJCC stage*						
IA	0.087	0.010‐0.788	ref.	—	ref.	—
IB	ref.		2.90	0.47‐18.04	0.0002	0.0001‐0.00057
IIA	2.08	0.68‐6.38	2.68	0.62‐11.66	1.12	0.46‐2.79
IIB	1.22	0.13‐11.43	2.62	0.20‐35.0	2.81	1.03‐7.63
IIC	7.69	2.31‐25.53	9.13	1.79‐46.69	5.86	1.77‐19.5
IIIA	1.66	0.55‐5.00	3.05	0.58‐16.06	4.62	1.38‐15.5
IIIB	2.49	0.89‐6.95	3.27	0.74‐14.48	8.17	1.39‐19.7
IIIC	3.28	1.12‐9.65	6.07	1.38‐26.74	21.9	9.00‐53.4
IVA	7.80	1.79‐34.00	7.68	1.68‐35.02	28.5	11.3‐71.5
IVB + C	10.16	3.68‐28.00	12.54	2.91‐53.79	36.8	15.5‐87.7
IVx	14.97	5.44‐41.19	15.11	3.49‐65.32	31.0	12.8‐75.1

Note

Adjusted for sex, race, primary site, grade, surgery information, and marital status.

Abbreviations: CI, confidence interval; CIF, cumulative incidence function; CSS, cancer‐specific survival; HR, hazard ratio; NEC, neuroendocrine carcinoma; OCT, other carcinoid tumor.

### 3.5 HRS for cancer‐specific mortality in NEC and OCT

What was more consistent between NEC and OCT was that compared with the reference, the trends of increasing HR estimates, showing significant differences with overlaps between adjacent categories in SEER historic stage (regional and distant), N status (N1a, N1b, N2a, and N2b), M status (M1a and M1b). Compared with M0, M1 had significant differences in CSS of NEC (HR = 4.80; 95% CI: 3.78‐6.10) and OCT (HR = 8.27; 95% CI: 5.89‐11.63), respectively. For NEC, compared with T1, no obvious differences in CSS were showed for any T stage. For OCT, all T stages had increasing HRs compared with T4a (N1b [HR = 3.49, 95% CI: 1.89‐6.45], T4b [HR = 7.39; 95% CI: 3.94‐13.86], and Tx [HR = 8.71; 95% CI: 4.57‐16.63]), except for T2 and T3. Compared with the reference, combined AJCC stages showed an obvious impact on CSS, with stages III and IV having increasing HRs (NEC: 4.93 and 13.75; OCT: 8.44 and 9.10), except for stage II (Table S2).

Advanced detailed 8th AJCC categories in NEC had increasing HRs with overlapping CIs (IIIC [HR = 6.07; 95% CI: 1.38‐26.74], IVA [HR = 7.68; 95% CI: 1.68‐35.02], IVB [HR = 12.54; 95% CI: 2.91‐53.79], and IVx [HR = 15.11; 95% CI: 3.49‐65.32]). Advanced detailed 8th AJCC categories in OCT showed increasing HRs with overlapping CIs (IIIA [HR = 4.62; 95% CI: 1.38‐15.5], IIIB [HR = 8.17; 95% CI: 1.39‐19.7], IIIC [HR = 21.9; 95% CI: 9.0‐53.4], IVA [HR = 28.5; 95% CI: 11.3‐71.5], and IV_B+C_ [HR = 36.8; 95% CI: 15.5‐87.7]). However, in the early and middle status of NEC and OCT, HRs did not increase with the increase of stages, or there was no difference in HRs in adjacent stages (Table 3).

## 4 DISCUSSION

NEN was a kind of uncommon tumor with high heterogeneity, with an increasing incidence in recent years.^10^, ^11^ Among them, pancreas and gastrointestinal sites were the most common locations of NEN, with an incidence of about 35.6 per million.^12^ According to different degrees of pathological differentiation, NENs were divided into well‐differentiated NETs, poorly differentiated NETs, and mixed neuroendocrine and/or non‐NETs with both adenocarcinoma and neuroendocrine components.^13^, ^14^, ^15^, ^16^ Because of great differences in biological behavior between NET and NEC, the 8th edition of the AJCC staging system focused on NETs, mainly aimed at NET, while NEC staging was based on the criteria of adenocarcinoma at the corresponding site.^17^, ^18^, ^19^, ^20^, ^21^ In this study, we evaluated the survival of NEC patients based on the 8th colorectal cancer staging system, including the analysis of patients with carcinoid tumor NOS and OCT.

The 8th edition AJCC staging system of colorectal cancer revised and added some of the details of regional lymph node (N) and distant metastasis (M) in anatomy, but had no update for T stages.^22^ Moreover, the 8th edition added the definition of stage M1c (peritoneal metastasis) on the basis of the 7th edition,^22^ because although peritoneal metastasis was only seen in 1% to 4% of colorectal cancer patients, the prognosis was much worse than that of patients with solid organ metastasis in stage M1a and stage M1b. However, the number of M1c patients in the SEER database was small, and the metastatic sites of some patients were not clearly recorded, thus M1c patients were included in M1b or M1 subgroup stage in our cohort. We found that significant differences in CSS were found with increasing N stage, M stage, and SEER historic stage in all three carcinoid tumors. Similar results were found from T2 to Tx (*P* < .001), except for T1 in all three carcinoid tumors, with 10‐year CSS of 88.0%, 44.4%, and 85.4% in each group, respectively. Why was the survival rate of pT1 more abnormal? This might be associated with the inherent nature of carcinoid itself.^16^, ^18^, ^19^, ^20^ According to the literature, T status of carcinoid generally needed to consider tumor size and invasive depth, but the 8th edition stage of colorectal cancer did not take into account the parameter of tumor size, although the tumor size was included in the T stage of NET staging system.^22^ Tumor size had been proved to be a significant parameter of metastasis in colorectal carcinoids.^11^, ^20^ Mani et al^23^ found the incidences of metastasis of tumor sizes with <1.0 cm, 1.0‐1.9 cm, and >2 cm were about 2%, 12%, and 70%, respectively. Schindl et al^24^ demonstrated that the tumor size of rectal carcinoid was associated with the status of N+ or M+. Therefore, it was conceivable that the prognosis of T1 with large diameter tumors might be worse than that of T2 with smaller tumors. Another reason might be different choices of treatments for pT1 tumors. It was generally accepted that tumors larger than 2 cm required radical surgery and associated lymphoid tissues should be dissected in order to look for possible lymph node metastases.^11^ However, the treatment of tumors smaller than 2 cm in diameter was controversial all the time.^19^ Although a metastatic rate of 4%‐30% was reported, for rectal carcinoid tumors with diameters between 1 and 2 cm, the metastatic status still could not be predictable.^25^ The guidelines bases on NETs from UKNET suggest that local resection was feasible for colorectal carcinoids less than 1 cm.^26^ However, based on the fact that N+ was also found in tumors less than 1 cm, these guidelines were opposed.^27^ Because the conservative treatment led to tumor spread, finally affecting the survival, it was bound to cause pT stage migration, so that colorectal carcinoid which should belong to stage T2 was misdiagnosed to the T1 stage using improper pathological diagnosis.

We also demonstrated that significant differences in CSS were found with increasing combined 8th AJCC stages (*P* < .001) in carcinoid tumor NOS, except for that between stage II and stage III (10‐year CSS rate: 82.6% vs 84.3%, *P* = .68), while combined 8th TNM stage in NEC and OTC showed greater separations in CSS despite continuous overlaps between groups. Maggard et al^28^ conducted an updated analysis of 11 427 patients with rectal carcinoids, and found a 5‐year survival rate of 87.5% for all stages. Kim et al^25^ reported the 5‐year actuarial overall survival rates for stages I, II, III, and IV were 100%, 80%, 51.4%, and 0%, respectively. The effect of deeper depth of invasion on prognosis might be greater than that of colorectal carcinoid patients with limitedly regional lymph node metastasis. This trend was more obvious in the results of the subgroup analysis of the detailed 8th AJCC carcinoid stage. However, in NEC and OCT, more highly malignant tumors might be included, whose biological behaviors were closer to that of adenocarcinoma, as reported by Yozu et al,^7^ thinking about that histologic and prognostic studies supported the reclassification of appendiceal Goblet cell carcinoids as Goblet cell adenocarcinoma, and its grade and stage were similar to those of colon adenocarcinoma.

Although combined 8th AJCC stage had a good ability to distinguish the 10‐year CSS of patients with NEC and OCT, detailed 8th AJCC stage did not seem to be applicable. Detailed 8th AJCC categories of advanced stages in all the three groups conferred increased HRs with overlapping CIs. However, in the early and middle status, HRs did not increase with the increase of stages, or there was no difference in HRs between adjacent stages. Similar grading prognostic results for carcinoid could be seen in lung carcinoid.^6^ These findings limited the usefulness of the 8th TNM staging system. Moreover, for all carcinoid tumors, N status seemed to have a higher prognostic value than T status and detailed 8th AJCC stage. More accurate staging methods of colorectal carcinoid tumor needed to be explored, particularly in sub‐stages from IIA to IIIB, to guide clinical treatment and prognosis in the future.

There were still some limitations and deficiencies in this study. First, although the included cases were mainly NEC and other poorly differentiated carcinoid types, partially well‐differentiated carcinoid tumors were still contained in the group of carcinoid tumor NOS, better staged according to the staging system of colorectal NET,^22^ finally affecting the survival statistics. Second, the information on tumor size, Ki‐67, and G1/2/3 status, were not clear in the SEER database, causing not accurately to classify malignant degrees of colorectal carcinoid. Third, a small number in some subgroups limited our estimation of survival in these groups. Finally, more studies suggested that molecular typing, such as gene mutations and abnormal expression profiles,^29^, ^30^ contributed to more accurate classification and prognosis of colorectal carcinoid, but these information was missing in the SEER database. Another issue was the classification heterogeneity of carcinoid tumors, the classifications of carcinoid tumor NOS, NEC, goblet cell carcinoid, mixed adenoneuroendocrine carcinoma, adenocarcinoid tumor NOS, enterochromaffin cell carcinoid, and atypical carcinoid tumor NOS were from SEER database standards, which might be related to the original pathological criteria of SEER data.

Despite these limitations, our study provided valuable information on the long‐term survival outcome of colorectal carcinoid, especially when the updated staging system had not been validated. Conclusively, based on the findings in this study, we proposed that the combined 8th TNM stage was not practical for judging the survival outcomes of colorectal carcinoid tumor NOS, especially in patients with stages II and III, but it provided useful prognostic information for NEC and OCT. For all carcinoid tumors, detailed 8th AJCC categories of advanced stages conferred increased HRs with overlapping CIs. However, in the early and middle status, HRs did not increase with the increase of stages, or there was no difference in HRs between adjacent stages. More optimized staging methods should be developed and validated in the future.

## CONFLICT OF INTEREST

The authors declare that they have no competing interests.

## AUTHOR CONTRIBUTIONS

XXL and ZQH conceived and designed the study, XKZ and WXH performed the analyses, PQG, CHC, and ZW provided assistance in writing the manuscript and support in interpreting results. All authors discussed the results and implications of the analysis and commented on the manuscript at all stages.

## Supporting information

Data S1

Figure S1

Table S1‐S2

## Data Availability

Research data are not shared.
