# Supplementary figures and images for: Prognostic significance for colorectal carcinoid tumors based on the 8th edition TNM staging system
Source: Cancer Med. 2020 Sep 8;9(21):7979–87. doi: 10.1002/cam4.3431 (PMC7643648; doi:10.1002/cam4.3431)

## Slide 1
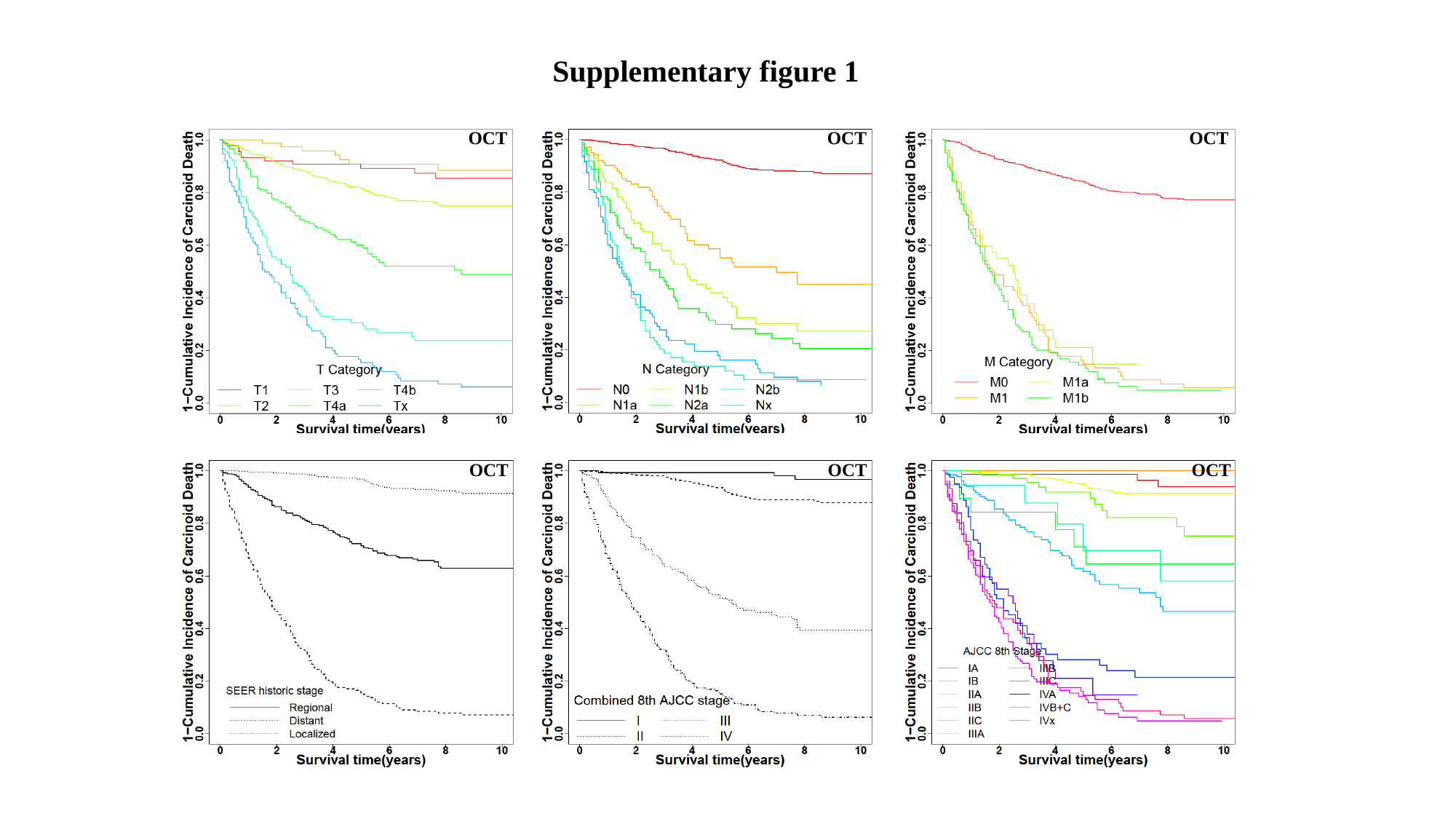

Supplementary figure 1
OCT
OCT
OCT
OCT
OCT
OCT

Supplement: Supplementary file 2 — Figure S1 [file CAM4-9-7979-s002.pptx]
