# Supplementary material for: Prognostic significance for colorectal carcinoid tumors based on the 8th edition TNM staging system
Source: Cancer Med. 2020 Sep 8;9(21):7979–87. doi: 10.1002/cam4.3431 (PMC7643648; doi:10.1002/cam4.3431)
Supplement: Supplementary file 3 — Table S1‐S2 [file CAM4-9-7979-s003.docx]

**Supplementary table 1. Demographic and Clinical Characteristics**

| **Characteristics** | **Patients, n (%)** | **Characteristics** | **Patients, n (%)** |
| --- | --- | --- | --- |
| Age (years, Mean±SD) | 58(±14.4) | Detailed 8^th^ AJCC stage |  |
| Year of diagnosis |  | IA | 372(8.7%) |
| 1988-2000 | 517(12.1%%) | IB | 181(4.2%) |
| 2001-2015 | 3769(87.9%) | IIA | 743(17.3%) |
| Sex |  | IIB | 88(2.1%) |
| Male | 2028(47.3%) | IIC | 49(1.1%) |
| Female | 2258(52.7%) | IIIA | 233(5.4%) |
| Race |  | IIIB | 744(17.3%) |
| White | 3404(79.4%) | IIIC | 338(7.9%) |
| Black | 616(14.4%) | IVA | 88(2.1%) |
| *Others | 266(6.2%) | IVB+C | 980(22.9%) |
| Primary Site |  | IVx | 470(11.0%) |
| Colon | 3598(84.0%) | SEER historic stage |  |
| Rectum | 688(16.0%) | Localized | 1052(24.5%) |
| Grade |  | Regional | 1661(38.7%) |
| I/II | 1416(33.0%) | Distant | 1573(36.8%) |
| III/IV | 1064(24.8%) | T stage |  |
| Unknown | 1806(42.2%) | T1 | 636(14.8%) |
| Surgery performed |  | T2 | 400(9.3%) |
| Yes | 3773(88.0%) | T3 | 1843(43.2%) |
| No | 513(12.0%) | T4a | 375(8.7%) |
| Marital status |  | T4b | 327(7.6%) |
| Married | 2494(58.3%) | Tx | 702(16.4%) |
| Divorced/separated | 456(10.6%) | N stage |  |
| Single | 743(17.3%) | N0 | 1548(36.1%) |
| ^#^Others | 593(13.8%) | N1a | 463(10.8%) |
| Histopathological type |  | N1b | 546(12.7%) |
| Carcinoid tumor NOS | 1726(40.2%) | N2a | 493(11.5%) |
| NEC | 1346(31.4%) | N2b | 549(12.8%) |
| OCT | 591(28.4%) | Nx | 687(16.1%) |
| Combined 8^th^ AJCC stage |  | M stage |  |
| I | 553(12.9%) | M0 | 2748(64.1%) |
| II | 880(20.5%) | M1a | 88(2.1%) |
| III | 1315(30.7%) | M1b | 980(22.8%) |
| IV | 1538(35.9%) | M1 | 470(11.0%) |

*Others: American Indian/AK Native, Asian/Pacific Islander and unknown ones. ^#^Others: unmarried or domestic partner, widowed and unknown ones. OCT: goblet cell carcinoid, mixed adenoneuroendocrine carcinoma, adenocarcinoid tumor, enterochromaffin cell carcinoid and atypical carcinoid tumor.

**Supplementary table 2. Adjusted CSS and Cumulative Incidence Function Regression Models**

| **TNM category** | **Carcinoid tumor NOS** | | **NEC** | | **OCT** | |
| --- | --- | --- | --- | --- | --- | --- |
|  | HR (CIF) | 95% CI | HR (CIF) | 95% CI | HR (CIF) | 95% CI |
| **SEER historic stage** |  |  |  |  |  |  |
| Localized | ref. | - | ref. | - | ref. | - |
| Regional | 3.38 | 1.64-6.98 | 2.99 | 1.51-5.95 | 2.80 | 1.70-4.60 |
| Distant | 8.96 | 3.21-25.09 | 7.80 | 3.92-15.50 | 8.11 | 3.47-18.98 |
| **Combined 8^th^ AJCC stage** |  |  |  |  |  |  |
| I | ref. | - | ref. | - | ref. | - |
| II | 3.37 | 0.97-11.76 | 1.97 | 0.75-5.14 | 2.45 | 0.84-7.15 |
| III | 2.09 | 0.51-8.66 | 4.93 | 2.01-12.11 | 8.44 | 2.53-28.24 |
| IV | 5.09 | 1.08-24.08 | 13.75 | 5.65-33.44 | 9.10 | 2.36-35.12 |
| **T stage** |  |  |  |  |  |  |
| T1 | ref. | - | ref. | - | ref. | - |
| T2 | 1.09 | 0.64-1.86 | 0.57 | 0.36-0.88 | 0.59 | 0.235-1.49 |
| T3 | 2.53 | 1.68-3.81 | 1.11 | 0.85-1.46 | 1.58 | 0.88-2.84 |
| T4a | 2.80 | 1.55-5.05 | 1.14 | 0.802-1.63 | 3.49 | 1.89-6.45 |
| T4b | 5.01 | 3.02-8.31 | 1.34 | 0.98-1.84 | 7.39 | 3.94-13.86 |
| Tx | 7.86 | 5.11-12.11 | 1.21 | 0.93-1.59 | 8.71 | 4.57-16.63 |
| **N stage** |  |  |  |  |  |  |
| N0 | ref. | - | ref. | - | ref. | - |
| N1a | 1.25 | 0.81-1.92 | 2.42 | 1.64-3.58 | 4.90 | 3.35-7.19 |
| N1b | 1.52 | 1.02-2.27 | 2.45 | 1.73-3.46 | 6.62 | 4.46-9.83 |
| N2a | 2.16 | 1.45-3.20 | 3.06 | 2.19-4.27 | 8.44 | 5.52-12.90 |
| N2b | 2.65 | 1.73-4.05 | 4.46 | 3.29-6.02 | 14.35 | 9.61-21.44 |
| Nx | 4.79 | 3.01-7.64 | 5.02 | 3.35-7.51 | 8.19 | 4.92-13.64 |
| **M stage** |  |  |  |  |  |  |
| M0 | ref. | - | ref. | - | ref. | - |
| M1a | 3.36 | 1.38-8.16 | 2.39 | 1.48-3.87 | 7.38 | 5.00-10.90 |
| M1b | 5.19 | 3.97-6.79 | 3.87 | 3.18-4.70 | 9.82 | 7.54-12.79 |
| M1 | 7.74 | 5.91-10.14 | 4.80 | 3.78-6.10 | 8.27 | 5.89-11.63 |

Note: Adjusted for sex, race, primary site, grade, surgery information and marital status. CIF, cumulative incidence function; HR, hazard ratio; CI, confidence interval.
